# Improving patient classification and biomarker assessment using Gaussian Mixture Models and Bayes’ rule

**DOI:** 10.18632/oncoscience.494

**Published:** 2019-12-23

**Authors:** Marina A. Guvakova

**Affiliations:** ^1^Department of Surgery, Division of Endocrine & Oncologic Surgery, Harrison Department of Surgical Research, Perelman School of Medicine, University of Pennsylvania, Philadelphia, PA

**Keywords:** cancer, probabilistic modeling, biomedical research

## Abstract

In clinical research, determining cutoff values for continuous variables in test results remains challenging, particularly when considering candidate biomarkers or therapeutic targets for disease. Distribution of a continuous variable into two populations is known as dichotomization and has been commonly used in clinical studies. We recently reported a new method for determining multiple cutoffs for continuous variables. The development of this original approach was based on fitting Gaussian Mixture Models (GMM) onto real-world clinical data. We also explored how to leverage Bayesian probability to minimize uncertainty while classifying individual patients into respective subpopulations. In addition, we investigated the performance of the proposed method for the distribution of classical prognostic markers in breast cancer. Finally, we applied the proposed method to analyze a candidate marker and a target for cancer therapy. Here, we present an overview of this method and our prospects for its implementation in biomedical and clinical research.

## INTRODUCTION

In the era of big-data-driven clinical research, numerous DNA, RNA, and protein measures are often collected concurrently from a myriad of patient samples using multiplexing technologies [1]. Despite these tremendous technological advancements, we have yet to determine how data obtained from diverse patient populations may enable the best possible treatment for individual patients. Creating probabilistic models of real-world data and incorporating them into the clinical decision-making process may provide this crucial answer. In the July 1, 2019 issue of Cancer Research, we introduced the original probabilistic classifier that takes into account the important consideration of individual patients and biomarkers classification in heterogeneous cancer [2].

## Gaussian Mixture Models (GMM) for patient classification

**Figure 1:**
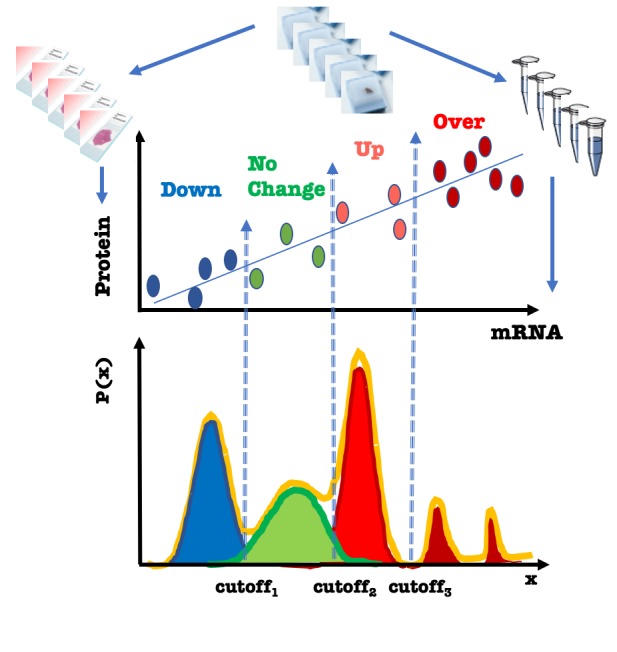
Lower scheme: GMM for multiclass classification with a set of cutoffs. X, continuous variable; P(x), the probability density function. Gaussians for overall (orange), similar-to-normal (green), downregulated (blue) and upregulated (red/dark red) variable in disease. Upper scheme, a proposed classification method assuming a correlation between the relative measurements of mRNA and protein in the same sets of samples (colored ovals).

In this paper, we present a step-by-step framework for using a GMM and Bayesian probability to minimize uncertainty and empower reasoning in classification of heterogeneous cancer. First, this method generates a probability distribution for all outcomes of a variable in a total patient population. It then provides an inference about the natural existence of a number of subpopulations with different outcomes independently of any predefined knowledge of that number (Figure1). The inference about each subpopulation’s characteristics is based on the concurrent analysis of datasets obtained from healthy and diseased samples, allowing for disease-specific outcome prediction. Next, this method estimates the proportion of patients in each subpopulation. Finally, it classifies individual patients into respective subpopulations using the Bayesian approach to probability. We have tested this classifier on mRNA data from hundreds of human breast tissue samples. However, when a correlation exists between gene mRNA levels and protein expression levels, protein measurement from the same sample could be used as a second criterion in classification (Figure1). Importantly, the classifier identifies patients with abnormally high or low levels of the molecular variable in their tumors. At the same time, it provides a means for exclusion of patients with no change in the levels of this variable in their tumors. Precise classification of patients is critical for targeted enrollment into clinical trials and for developing personalized therapies to improve patient outcomes. It is likely that application of the GMM approach can help meet the outstanding need for more personalized care for each patient.

## GMM for biomarker research

Importantly, our method is expected to be more precise than traditional statistical methods in the clinical validation of biomarkers. Biomarkers are powerful tools for identifying a variety of health conditions and diseases and monitoring responses to therapies. Development of biomarkers is limited by difficulty in determining accurate cutoff values for diseases, however. Candidate biomarkers are often separated using a single cutoff point that defines subpopulations with low and high biomarker levels. These single cutoff points are often values that have already been published, specific sample values (such as the median), or “optimized” cutoffs, which correlate with clinical test and survival data [3]. However, there is growing concern about the natural existence of single cutoffs and their accuracy for a given variable in disease diagnosis [4, 5]. To improve biomarker assessment, we propose the GMM-based method that provides an effective approach for calculating multiple cutoffs with confidence intervals. Through the output of a probability of all possible outcomes of the variable, this method handles missing data and extracts much more information from small datasets. It may, therefore, save time and money by reducing costs incurred by misclassification. As a result of the general flexibility of the GMM approach, this classifier can also be deployed to analyze a large number of biomarker datasets currently awaiting assessments of clinical value.

## CONCLUSION

Our new probabilistic classifier addresses the common problem of cutoff determination based on a continuous variable and provides a “first-aid” solution for resolving ambiguity of a biomarker or a therapeutic target in disease. Additionally, it could evolve further into an automated technology used to determine target populations and validate biomarkers. This, in turn, could deliver automated inferences to personalized healthcare.
